# The unique alterations of hippocampus and cognitive impairment in chronic obstructive pulmonary disease

**DOI:** 10.1186/1465-9921-14-140

**Published:** 2013-12-21

**Authors:** Jing Li, Guang-He Fei

**Affiliations:** 1 Pulmonary Department, First Affiliated Hospital of Anhui Medical University, Hefei, Anhui 230022, China

**Keywords:** Chronic obstructive pulmonary disease, Cognitive impairment, Hippocampus, Pulmonary function, S100B

## Abstract

**Background:**

Cognitive impairment has been found in chronic obstructive pulmonary disease (COPD) patients. However, the structural alteration of the brain and underlying mechanisms are poorly understood.

**Methods:**

Thirty-seven mild-to-moderate COPD patients, forty-eight severe COPD patients, and thirty-one control subjects were recruited for cognitive test and neuroimaging studies. Serum levels of S100B,pulmonary function and arterial blood gas levels were also evaluated in each subject.

**Results:**

The hippocampal volume was significantly smaller in COPD patients compared to the control group. It is positively correlated with a mini mental state examination (MMSE) score, SaO_2_ in mild-to-moderate COPD patients, the levels of PaO_2_ in both mild-to-moderate and severe COPD patients. Higher S100B concentrations were observed in mild-to-moderate COPD patients, while the highest S100B level was found in severe COPD patients when compared to the control subjects. S100B levels are negatively associated with MMSE in both mild-to-moderate and severe COPD patients and also negatively associated with the hippocampal volume in the total COPD patients.

**Conclusions:**

Hippocampal atrophy based on quantitative assessment by magnetic resonance imaging does occur in COPD patients, which may be associated with cognitive dysfunction and the most prevalent mechanism accountable for hippocampal atrophy is chronic hypoxemia in COPD. Higher serum S100B levels may be peripheral biochemical marker for cognitive impairment in COPD.

## Background

Chronic obstructive pulmonary disease (COPD) is a primary airway inflammatory disease characterized by irreversible airflow limitation which results in hypoxemia and hypercapnia. Meanwhile, it is also realized as a complex multi-component disorder [1-4]. Cognitive impairment has been found as one of the important extrapulmonary manifestation in patients with COPD [5-8]. In our previous study, we also found that cognitive dysfunction is associated with the classification of disease severity [9]. However, whether some structural brain abnormalities are associated with poor cognitive performances in COPD patients has not been fully explored.

The hippocampus which is located inside the medial temporal lobe is a major component of the brain. It contains two main interlocking parts (Ammon’s horn and the dentate gyrus) and four histological divisions (cornu ammonis 1 (CA1), CA2, CA3 and CA4). It plays a key role in cognitive function and is particularly vulnerable to the adverse effects of hypoxemia. As morphologic evidence, magnetic resonance imaging (MRI) studies demonstrated that hippocampal atrophy is a diagnostic biomarker for cognitive impairment [10,11]. Lower hippocampal volume detected by MRI was a consistent finding in mild cognitive impairment (MCI) and Alzheimer’s disease [12,13]. To our knowledge, quantitative assessment of the hippocampal region in COPD patients has not yet been carried out, although there may be a pattern of cognitive impairment specific to COPD patients [8]. Therefore, the question arises as to whether detectable structural changes of hippocampal volume occur in COPD patients.

S100B, a member of the S100 protein family, is a calcium-binding protein [14]. It is a brain derived protein implicated in CNS function generally and the hippocampus in particular [15]. Increased S100B levels are observed in patients suffering from chronic neurodegenerative disorders such Alzheimer’s disease [16-18]. Furthermore, it is also studied as a peripheral biochemical marker for cognitive ability [19]. Previous studies showed that serum S100B levels were negatively correlated with MMSE scores [20]. However, little data exists with regards to this biomarker and cognitive performances in COPD patients.

In this study, we evaluated the hippocampal volumes and cognitive performances in COPD patients at different stages. In order to explore the possible mechanisms involved in this pathology, we also investigated the associations between the hippocampal volume, pulmonary function parameters and arterial blood gases in COPD patients.

## Material and methods

### Subjects

Eighty-five subjects, including thirty-seven mild-to-moderate COPD patients, forty-eight severe COPD patients, and thirty-one control subjects participated in the present study. The three groups were matched for age, sex, education level, body mass index (BMI), smoking status, and cardiovascular disease. The COPD patients came from the First Affiliated Hospital of Anhui Medical University. The diagnoses and classification of COPD were made according to the Global Initiative for Chronic Obstructive Lung Disease (GOLD) 2011 guidelines [21]. All patients were clinically stable for ≥8 weeks and were treated only with necessary medications: antibiotics, β2 adrenoreceptor agonists, ambroxol, and oxygen therapy. Exclusion criteria were: Dementia, sleep disorders, obstructive sleep apnoea (OSA), chronic kidney disease, liver disease, head injury, psychiatric disorders (depression, anxiety disorders, schizophrenia, or alcohol abuse), and usage of any drugs which might affect cognitive performance. All participants were assessed by a complete physical examination by three raters including a respiratory physician, a cardiologist, and a neuropsychologist. All subjects had given written informed consent to participate and were told of the possible risks in the study; the protocol was approved by the Human Investigation Committee of Anhui Medical University. The experiment was performed from November 1, 2011 to January 15, 2013.

### Arterial blood gas analysis

Arterial blood gas analysis was performed by using Stat Profile Critical Care Xpress (Nova Biomedical, Waltham, Mass., USA). The arterial oxygen tension (PaO_2_), arterial carbon dioxide tension (PaCO_2_) and blood oxygen saturation (SaO_2_) was evaluated for all subjects while they breathed room air in the supine position.

### Pulmonary function tests

Standardized pulmonary function tests were performed using a dry spirometer device (Erich Jaeger GmbH, Hoechberg, Germany). After inhaling salbutamol (Ventolin; GlaxoSmithKline; London, UK), the forced vital capacity (FVC), the forced expiratory volume in one second (FEV_1_), and FEV_1_/FVC ratio were obtained. In the subjects with FEV_1_/FVC < 0.70, FEV_1_ ≥ 80% predicted were classified as mild COPD; 50% ≤ FEV_1_ < 80% predicted were moderate COPD; 30% ≤ FEV_1_ < 50% predicted were severe COPD and FEV_1_ < 30% predicted were very severe COPD. Mild and moderate COPD patients were defined as mild-to-moderate COPD group, while severe and very severe COPD patients were defined as severe COPD group [21].

### Cognitive ability tests

Cognitive function was measured using the MMSE Test, which is the most commonly used screening test for cognitive impairment. This instrument explores spatial and temporal orientation, short- and long-term verbal memory, attention, verbal attention, and practical ability in 12 items and 30 questions. The correct answers to one question gives 1 score point (total from 0 to 30). A score less than 24 indicated a mild cognitive impairment. All examinations were performed by a trained neuropsychologist.

### Measurement of serum S100B concentration

Blood samples (3 ml) were collected by venipuncture with a tube without anticoagulants around 6.30 a.m. in a regular exanimation. Serum was obtained by centrifugation at 3000 rpm for 10 min, and then frozen at −70°C until studied. Serum S100B concentrations were measured using a commercial enzyme-linked immunosorbent assay (ELISA) kit (RD192090100R, BioVendor, Brno, Czech Republic) according to the manufacturer’s instructions. The intra-assay and inter-assay variation coefficients were 3.3% and 7.7%, respectively. The assay standard range is 0.05-2 ng/ml. The limit of detection is 15 pg/ml.

### Magnetic resonance imaging

All MRI studies were performed on a 3.0-Tesla superconducting system (Trio, Tim, VB15, Sciemens, Erlangen, Germany). T1-weighted MRI scans were collected using a three dimensional (3D) magnetization prepared rapid gradient echo (MP-RAGE) sequence for each subject, using the following parameters: TE/TR = 5.5/1.5; flip angle = 30; matrix size = 256 × 256; slice thickness = 1.4 mm; slices = 248. The total scan time was 2 minutes and 56 seconds.

### Volumetric measurement

Volumetric measurements were performed with a semiautomated software package in the Department of Radiology, First Affiliated Hospital of Anhui Medical University. The MRI images were transferred to ADM workstation (version 4.4). In this study, the contours of hippocampus were manually outlined by two experienced raters blinded to participants’ age, sex and diagnosis to insure consistency. Boundary definition for hippocampus was according to well-established protocol [22]. Relevant images from standard atlases were referred to ensure a consistent reference to the boundaries and landmarks for hippocampus. Once the outline of the hippocampus had been defined, a slice volume was calculated by multiplying the area outlined by slice thickness. The total hippocampal volume was then calculated by adding the slice volumes. The interrater reliability of the volume measurements was determinate by the intraclass correlation coefficients (ICC) [23]. The ICC for the right and left hippocampus were 0.96 and 0.97, respectively. Normalization of the original hippocampal volume with intracranial volume (ICV) was carried out using the method described by Jack CR Jr et al. [24].

### Statistical analysis

Categorical variables were compared using a chi-squared test. Statistical significance of the differences between mean values was tested using one-way analysis of variance for variables with normal distribution and a Kruskal-Wallis test otherwise. A Bonferroni correction for multiple comparisons was applied. The Pearson Correlation analyses were used to verify the relationship between numerical variables with normal distribution, while the Spearman correlation test was applied to nonparametric variables. All statistical analyses were performed using Graphpad Prism5. Values of p < 0.05 were considered to be significant.

## Results

### Demographic characteristics in the control group and COPD groups

Demographic characteristics of the control, the mild-to-moderate and the severe COPD groups are depicted in Table 1. The three groups were statistically similar with respect to age, sex, smoking, education level, BMI and cardiovascular disease (P > 0.05).

**Table 1:** Demographic characteristics in the control group and mild-to-moderate and severe COPD groups

**Characteristic**	**Control group (n = 31)**	**Mild-to-moderate COPD group (n = 37)**	**Severe COPD group (n = 48)**	***P*** **Value**
Age, years	66.48 ± 6.96	69.27 ± 8.08	67.60 ± 7.63	0.31^a^
Females/males, n	10/21	9/28	13/35	0.76^b^
Smoking status, %				0.91^b^
Never smoked	41.93	35.14	45.83	
Current smoker	25.81	29.73	25.00	
Ex-smoker	32.26	35.13	29.17	
Cigarettes smoked, pack-years				
Current smoker	34.13 ± 12.11	33.09 ± 8.11	34.13 ± 5.52	0.95^a^
Ex-smoker	27.25 ± 8.68	29.00 ± 6.60	32.64 ± 7.81	0.22^a^
Education, years	8.07 ± 4.97	9.49 ± 4.75	9.42 ± 4.01	0.35^a^
BMI, kg/m^2^	22.51 ± 2.67	22.11 ± 3.66	21.91 ± 3.29	0.73^a^
Cardiovascular disease, %	38.71	54.05	54.17	0.34^b^

Data are presented as means ± SD, except where indicated otherwise. Cardiovascular diseases include hypertension, coronary artery disease and congestive heart failure. BMI = Body Mass Index.

^a^: One-way analysis of variance. ^b^: Analysis of Chi-Square test.

### The alterations of the hippocampal volumes and serum S100B level in COPD patients

**Figure 1:**
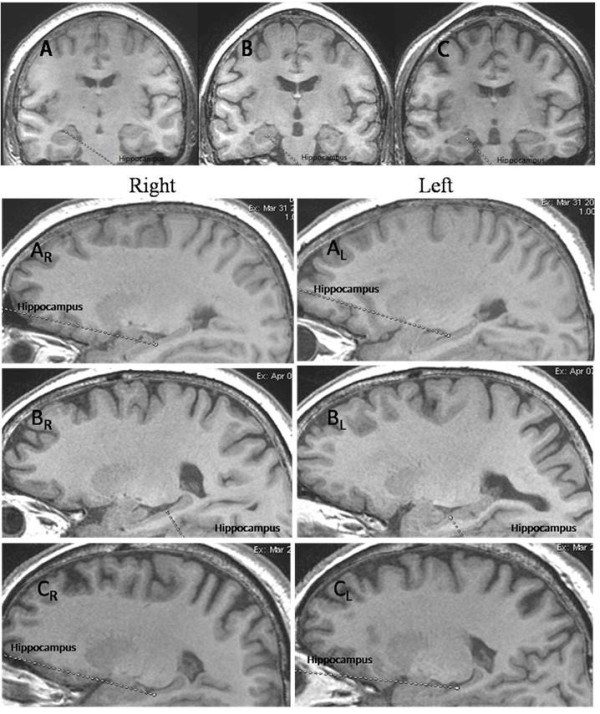
**MRI images from a control subject, a mild-to-moderate COPD patient and a severe COPD patient.** A line has been placed over the region of the hippocampus in one hemisphere. A = control subject; B = mild-to-moderate COPD patient; C = severe COPD patient; A_R_ = the right hippocampus of control subject; A_L_ = the left hippocampus of control subject; B_R_ = the right hippocampus of mild-to-moderate COPD patient; B_L_ = the left hippocampus of mild-to-moderate COPD patient; C_R_ = the right hippocampus of severe COPD patient; C_L_ = the left hippocampus of severe COPD patient.

As shown in Figure 1, hippocampal atrophy was seen in the mild-to-moderate and severe COPD patients when compared with control subjects.

**Figure 2:**
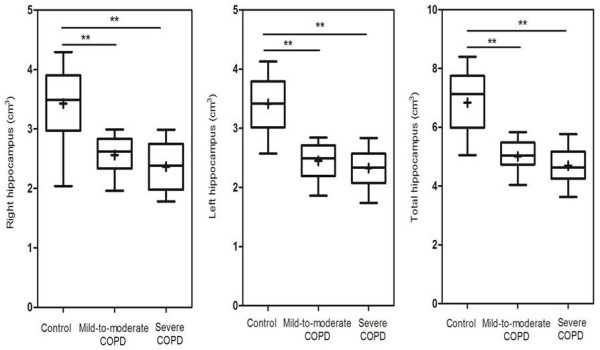
**Box plot of the hippocampal volumes in control group and mild-to-moderate and severe COPD groups.** The horizontal line through each box represents the median. The ends of each box represent the 25^th^ and 75^th^ percentile locations, and the lines represent the range of the date. The cross of each box represents the mean.* * P < 0.01.

Table 2 and Figure 2 show that both the right and left hippocampal volumes were significantly smaller in the mild-to-moderate (right P < 0.01, left P < 0.01) and severe COPD (right P < 0.01, left P < 0.01) groups compared to the control group. However, there was no significant difference between the mild-to-moderate COPD group and severe COPD group (P > 0.05).

**Table 2:** Clinical characteristics, hippocampal volume and serum S100B concentration in the control and COPD groups

**Test parameter**	**Control group (n = 31)**	**Mild-to-moderate COPD group (n = 37)**	**Severe COPD group (n = 48)**	***P*** **Value**
FEV_1_, % predicted	106.10 ± 15.14	61.14 ± 6.35^*^	34.15 ± 8.89^*,+^	<0.01
FVC, % predicted	94.85 ± 14.40	76.80 ± 10.42^*^	54.13 ± 14.93^*,+^	<0.01
FEV_1_/ FVC	90.07 ± 7.97	61.89 ± 6.27^*^	49.85 ± 10.47^*,+^	<0.01
PaO_2_, mmHg	91.82 ± 6.95	71.89 ± 6.94^*^	66.36 ± 11.83^*^	<0.01
PaCO_2_, mmHg	37.00 ± 2.72	40.27 ± 6.53	48.27 ± 10.61^*,+^	<0.01
SaO_2_, %	97.50 (97.00-97.90)^a^	92.15 ± 2.86^*^	90.45 (84.80, 93.73)^a,*^	<0.01^b^
Rt hippocampus, cm^3^	3.43 ± 0.59	2.56 ± 0.31^*^	2.37 ± 0.38^*^	<0.01
Lt hippocampus, cm^3^	3.41 ± 0.47	2.45 ± 0.29^*^	2.33 ± 0.29^*^	<0.01
Tl hippocampus, cm^3^	6.84 ± 1.01	5.01 ± 0.49^*^	4.69 ± 0.57^*^	<0.01
ICV, cm3	1323.84 ± 112.37	1296.85 ± 111.35	1280.47 ± 130.17	>0.05
MMSE score	28.00(27.00-29.00)^a^	24.57 ± 2.24^*^	22.15 ± 2.88^*,+^	<0.01^b^
S100B, pg/ml	60.88 ± 8.43	77.02 ± 9.72^*^	105.70 ± 13.30^*,+^	<0.01

Data are presented as means ± SD. *P* value: One-way analysis of variance, except where indicated otherwise. ^*^P <0.01 vs. control group; ^+^*P* < 0.01 vs. mild-to-moderate group. ^a^: Medians (interquartile range). ^b^: Kruskal-Wallis test.

FEV_1_ = forced expiratory volume in one second; FVC = forced vital capacity; PaO_2_ = arterial oxygen tension; PaCO_2_ = arterial carbon dioxide tension; SaO_2_ = blood oxygen saturation; MMSE = mini mental state exam; Rt = right; Lt = left; Tl = total; ICV = intracranial volume.

Table 2 also shows that the serum S100B levels increased significantly in the mild-to-moderate (P < 0.01) and severe COPD (P < 0.01) groups compared with the control group. Furthermore, the serum S100B concentration was significantly higher in the severe COPD group than that in the mild-to-moderate COPD group (P < 0.01).

### The cognitive impairment in COPD patients

Table 2 shows that the MMSE score was significantly lower in the mild-to-moderate (P < 0.01) and severe COPD (P < 0.01) groups compared with the control group. Moreover, the MMSE score was significantly lower in the severe COPD group than in the mild-to-moderate COPD group (P < 0.01).

### Correlations between hippocampal volumes and relevant factors

**Figure 3:**
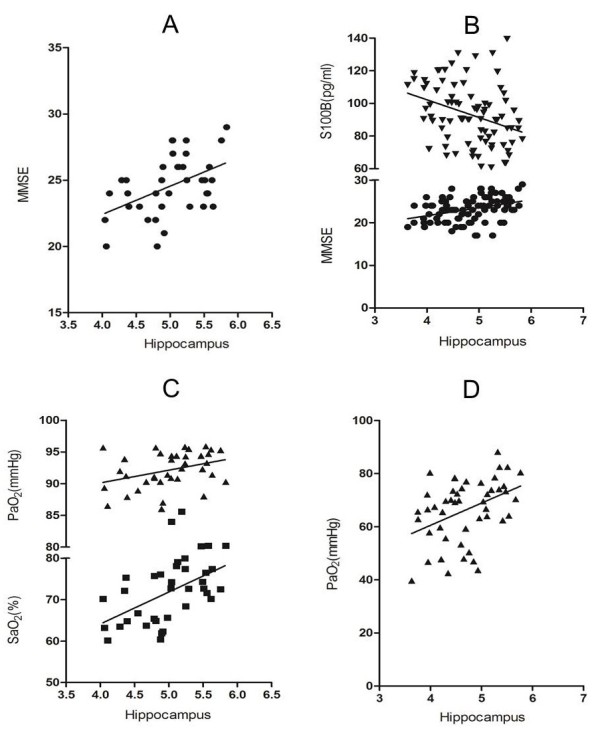
**Pearson correlations between the volumes of hippocampus and relevant factors in COPD groups.** ● = MMSE scores; ▼ = serum S100B concentrations; ▲ = PaO_2_; ▲ = SaO_2_. **A** = mild-to-moderate COPD group: MMSE (r = 0.47, P < 0.01). **B** = total COPD patients: MMSE (r = 0.36, P < 0.01); S100B (r = −0.33, P < 0.01). **C** = mild-to-moderate COPD group: PaO_2_ (r = 0.55, P < 0.01); SaO_2_ (r = 0.35, P < 0.05). **D** = severe COPD group: PaO_2_ (r = 0.40, P < 0.01).

Table 3 shows the correlation between the hippocampal volume, pulmonary function parameters, arterial blood gases, MMSE scores and serum S100B levels in COPD patients. The hippocampal volume was positively correlated with the MMSE scores, PaO_2_ and SaO_2_ (r = 0.47, P < 0.01; r = 0.55, P < 0.01; r = 0.35, P < 0.05, respectively) (Figure 3A and Figure 3C) in the mild-to-moderate COPD group, while it was positively associated with PaO_2_ (r = 0.46, P < 0.01) in the severe COPD group (Figure 3D). Furthermore, it was also positively associated with the MMSE scores (r = 0.36, P < 0.01) and negatively correlated with S100B levels (r = −0.33, P < 0.01) in the total COPD patients (Figure 3B).

**Table 3:** Relationships between the relative test values and hippocampal volumes in the groups with COPD of different severity

**Test parameter**	**Mild-to-moderate COPD group hippocampal volume (n = 37)**		**Severe COPD group hippocampal volume (n = 48)**		**Total COPD group hippocampal volume (n = 85)**	
	**r**	***P***	**r**	***P***	**r**	***P***
PaO_2_	0.55	**<0.01**	0.40	**<0.01**	0.51^a^	**<0.01**
PaCO_2_	0.15	NS	−0.14	NS	−0.15^a^	**NS**
SaO_2_	0.35	**<0.05**	0.19^a^	NS	0.29^a^	**<0.01**
FVC	0.13	NS	0.01	NS	0.21	**<0.05**
FEV_1_	0.14	NS	−0.12	NS	0.22^a^	**<0.05**
FEV_1_/FVC	−0.05	NS	−0.09	NS	0.10^a^	NS
MMSE	0.47	**<0.01**	0.19	NS	0.36	**<0.01**
S100B	−0.24	NS	−0.15	NS	−0.33	**<0.01**

FEV_1_ = forced expiratory volume in one second; FVC = forced vital capacity; PaO_2_ = arterial oxygen tension; PaCO_2_ = arterial carbon dioxide tension; SaO_2_ = blood oxygen saturation; MMSE = mini mental state exam; NS = no significant correlations. r = Pearson Correlation coefficient (except where indicated otherwise); ^a^: Spearman rank correlation coefficient.

### Correlations between cognitive function and relevant factors

**Figure 4:**
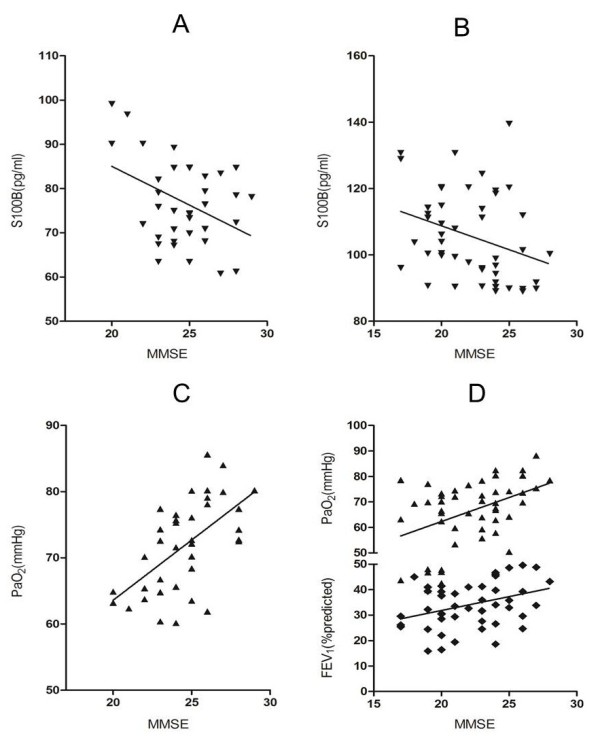
**Pearson correlations between MMSE scores and relevant factors in COPD groups.** ▼ = serum S100B concentrations; ▲ = PaO_2_; ■ = FEV_1_% predicted. **A** = mild-to-moderate COPD group: S100B (r = −0.40, P < 0.01); **B** = severe COPD group: S100B (r = −0.31, P < 0.01); **C** = mild-to-moderate COPD group: PaO_2_ (r = 0.59, P < 0.01); **D** = severe COPD group: PaO_2_ (r = 0.46, P < 0.01); FEV_1_ (r = 0.35, P < 0.05).

Table 4 shows the correlation between MMSE scores, pulmonary function parameters, arterial blood gases and the concentrations of serum S100B in COPD patients. The MMSE score was positively associated with PaO_2_ (r = 0.59, P < 0.01) and negatively correlated with serum S100B concentration ((r = −0.40, P < 0.05) in the mild-to-moderate COPD group, while it was positively associated with PaO_2_, SaO_2_ and FEV_1_ (r = 0.46, P < 0.01; r = 0.37, P < 0.01; r = 0.35, P < 0.05, respectively) and negatively correlated with S100B (r = −0.31, P < 0.05) in the severe COPD group (Figure 4).

**Table 4:** Relationships between the relative test values and MMSE score in the groups with COPD of different severity

**Test parameter**	**Mild-to-moderate COPD group MMSE (n = 37)**		**Severe COPD group MMSE (n = 48)**		**Total COPD group MMSE (n = 85)**	
	**r**	***P***	**r**	***P***	**r**	***P***
PaO_2_	0.59	**<0.01**	0.46	**<0.01**	0.54^a^	**<0.01**
PaCO_2_	0.12	NS	−0.11	NS	−0.16^a^	NS
SaO_2_	0.21	NS	0.37^a^	**<0.01**	0.36^a^	**<0.01**
FVC	0.18	NS	0.17	NS	0.39	<**0.01**
FEV_1_	0.25	NS	0.35	**<0.05**	0.49^a^	**<0.01**
FEV_1_/FVC	0.03	NS	0.13	NS	0.31^a^	**<0.05**
S100B	−0.40	<**0.05**	−0.31	<**0.05**	−0.52	**<0.01**

FEV_1_ = forced expiratory volume in one second; FVC = forced vital capacity; PaO_2_ = arterial oxygen tension; PaCO_2_ = arterial carbon dioxide tension; SaO_2_ = blood oxygen saturation; MMSE = mini mental state exam; NS = no significant correlations. r = Pearson Correlation coefficient (except where indicated otherwise); ^a^: Spearman rank correlation coefficient.

## Discussion

In the present study, we measured hippocampal volume and found hippocampal atrophy by MRI in COPD patients. This observation shows for the first time that structural alteration of the hippocampus may be involved in the cognitive impairment in COPD. Furthermore, we evaluated the serum S100B concentration and found that it is higher in COPD patients compared with that in control subjects.

Structural magnetic resonance imaging is the most clinical approach used to detect brain abnormalities in individuals who might be at risk for Alzheimer’s disease and MCI [12,25,26]. Recent studies have reported that hippocampal atrophy can be used as a biomarker for cognitive dysfunction and the rate of hippocampal atrophy was significantly correlated with the cognitive function in Alzheimer’s disease [13]. To our knowledge, quantitative assessment of the hippocampal region in COPD patients has not yet been carried out. In our previous study, we observed an association between cognitive decline and the classification of severity during disease progression in COPD patients [9]. In the present study, we further attempted to find morphological evidence that accounts for cognitive impairment in COPD patients. Therefore, we measured hippocampal volume in patients with different severity of COPD. Surprisingly, we found that hippocampal atrophy did occur in COPD patients and there were significant correlations with PaO_2_, SaO_2_ and MMSE which may account for cognitive decline in COPD patients. However, the mechanism is poorly understood. One possible explanation is that COPD patients exist in a state of low-grade systemic inflammation [3] and persistent airflow limitation which leads to chronic hypoxemia.

Chronic hypoxemia is a major mechanism that can adversely affect cognitive performance and hippocampal volume [27-30]. In this study, we also examined the relationships between hippocampal volume and relevant factors in mild-to-moderate and severe COPD groups, respectively. We found that hippocampal volume was positively correlated with the level of PaO_2_ in both mild-to-moderate and severe COPD groups. In addition, it was positively associated with SaO_2_ in mild-to-moderate COPD group. These findings show that the chronic hypoxemia in COPD patients may induce hippocampal atrophy, which plays a crucial role in cognitive impairment.

Interestingly, our present findings show that there were no significant differences of hippocampal volumes between mild-to-moderate and severe COPD groups. One possible explanation is that the cumulative loss of neurons and their connections results in hippocampal atrophy in the early stage of the disease, while the structural change converts to the functional deterioration in the later stage.

In addition, as we investigated the associations between hippocampal volume and cognitive function in COPD groups, we found that the hippocampal volume is positively correlated with cognitive ability in mild-to-moderate COPD patients. However, this was not shown in the severe COPD group. One possible explanation is that other regions of brain, such as transentorhinal, parahippocampal gyrus and entorhinal cortexes, which also play a critical role in the neural control of cognitive function [31], may also be affected by hypoxemic insults and systemic inflammatory stress with the progress of the disease. Therefore, there was no lineary correlation between hippocampal atrophy and cognitive impairment in the later stage of the disease.

S100B is a 21 kDa calcium binding protein produced and released primarily by astrocytes [32], which has been investigated as a biochemical marker of central nervous system injury. Previous studies have demonstrated that S100B could exert neurotrophic or neurotoxic effects depending on its concentration. High S100B concentration can be detrimental. It has been proposed that S100B may be the “CRP (C-reactive protein) of the brain” [14]. Some previous studies have showed that the levels of serum S100B were significant higher in patients with obstructive sleep apnea syndrome (OSAS) or Alzheimer’s disease [16,33]. In the present study, we evaluated the serum S100B concentration and found that it is higher in COPD patients compared with that in control subjects. One possible explanation is that changes in hippocampal structure and volume appear to arise from a reduction in neural processes and synapses, rather than from neuronal loss. However, future studies are needed to address the precise mechanisms underlying this observation.

Finally, we observed an association between cognitive decline and the classification of severity during disease progression in COPD patients and the level of PaO_2_ was positively correlated with cognitive ability. This observed cognitive impairment and relevant factors in COPD are in agreement with our previous report [9]. These findings suggest that the chronic hypoxemia plays a key role in the cognitive impairment in COPD patients.

A limitation of this study is the relatively small size of the participant population, which may partially account for the weak association between some of the measurements in our results. While the number of cases will be continuously increased in our future study, we anticipate that this study will lead to the realization of cognitive impairment and some structural abnormalities of brain in COPD patients in the field. Second, other brain regions, especially the entorhinal cortex, transentorhinal and parahippocampal gyrus which also play a critical role in the neural control of cognitive function need to be examined. Third, the MMSE, which has proven useful and sufficient for cognitive measurements, provides a broad scale and does not include measures of executive function. A neuropsychological testing battery will have to be considered in future studies to provide detailed analysis and quantification of cognitive functions of patients. Nonetheless, our results, at least preliminarily, suggest that neuroimaging studies may be useful in identifying COPD patients who are at increased risk of cognitive impairment.

Given the significant clinical implications of this study, further studies are needed to gain much more insight into this problem. The application of neuropsychological tests which is designed for quantifying specific parameters of certain mental function, such as visual memory, verbal fluency, and visuospatial deserve testing in COPD. Meanwhile, some brain regions, such as the entorhinal cortex, transentorhinal and parahippocampal gyrus critical for cognitive function need further explorations.

## Conclusions

In conclusion, hippocampal atrophy which leads to cognitive dysfunction does occur in COPD. MRI based quantitative assessment of the hippocampus provides direct evidence that COPD patients exhibit cognitive impairment. The most prevalent mechanism accountable for hippocampal atrophy in COPD patients is chronic hypoxemia. Higher serum S100B level may be the peripheral biochemical marker for cognitive impairment in COPD.

## Abbreviations

COPD: Chronic obstructive pulmonary disease; MMSE: Mini mental state examination; MRI: Magnetic resonance imaging; FVC: Forced vital capacity; FEV1: Forced expiratory volume in one second.

## Competing interests

The authors declare that they have no competing interests.

## Authors’ contributions

JL participated in study design, data acquisition and analysis and drafted the manuscript; G-HF participated in study design and coordination and critically revised drafts of the manuscript. All authors read and approved the final manuscript.
